# Colobome chorioretinien bilateral: à propos d’un cas

**DOI:** 10.11604/pamj.2018.30.261.15990

**Published:** 2018-08-07

**Authors:** Seydou Diallo, Seydou Bakayoko, Brainima Coulibaly, Mohamed Kole Sidibe, Nouhoum Guirou

**Affiliations:** 1 Institut d’Ophtalmologie Tropicale de l’Afrique, Bamako, Mali

**Keywords:** Colobome choriorétinien, bilatéral, enfant, Chorioretinian coloboma, bilateral, child

## Abstract

La présence d'un colobome choriorétinien soulève souvent des problèmes cliniques entraînant parfois une certaine confusion. Néanmoins, le diagnostic se doit d'être aussi précis que possible pour plusieurs raisons. L'association de certaines anomalies congénitales de la papille avec d'autres pathologies neurologiques endocriniennes ou systémiques et le problème de diagnostic différentiel avec d'autres pathologies ophtalmologiques. Nous rapportons un cas clinique d'un enfant âgé de 6 ans, le premier garçon d'une famille de 2 enfants présentant un colobome papillaire bilatéral sans d'autres anomalies malformatives associées. Dans ses antécédents: sont accouchement s'est déroulé normalement et sont statut vaccinal été à jour. L'examen ophtalmologique de son père et de sa petite sœur âgée de 4 ans été sans particularité, cependant sa mère présente un strabisme divergent à l'œil droit.

## Abstract (en)

Chorioretinian coloboma often raises clinical issues leading sometimes to some confusion. Nevertheless, diagnosis must be as accurate as possible for several reasons: the association of some congenital anomalies of the papilla with other neurologic, endocrine and systemic disorders as well as issues in differential diagnosis between it and other ophthalmic disorders. We report a clinical case of a 6 year old child, the first boy in a family with 2 children, with bilateral papillar coloboma and without other associated malformations. Patient?s history: normal birth and updated immunisation status. His father and his 4-year-old sister underwent ophthalmic examination which was unremakable. However his mother had diverge strabismus in the right eye.

## Introduction

Les colobomes, dits « typiques », résultent d´une anomalie de la fermeture de la fissure embryonnaire qui peut se produire à n´importe quel niveau d´une ligne inféronasale étendue de la papille en arrière à la pupille en avant. La fermeture de la fissure, qui débuterait vers la sixième semaine, commencerait au milieu de celle-ci pour s´étendre simultanément en direction de la papille et de l´iris. Au niveau du colobome, il y a absence de différenciation de la rétine, de l´épithélium pigmenté et par conséquent de la choroïde. En général, la sclère est recouverte d´une mince couche de rétine indifférenciée et transparente expliquant l´aspect typiquement blanc de ces colobomes [1] toute les structures de l'œil peuvent être touchées [2]. Les formes atteignant le segment postérieur sont de mauvais pronostic [3]. Nous présentons un cas de colobome choriorétinien bilatéral.

## Patient et observation

**Figure 1:**
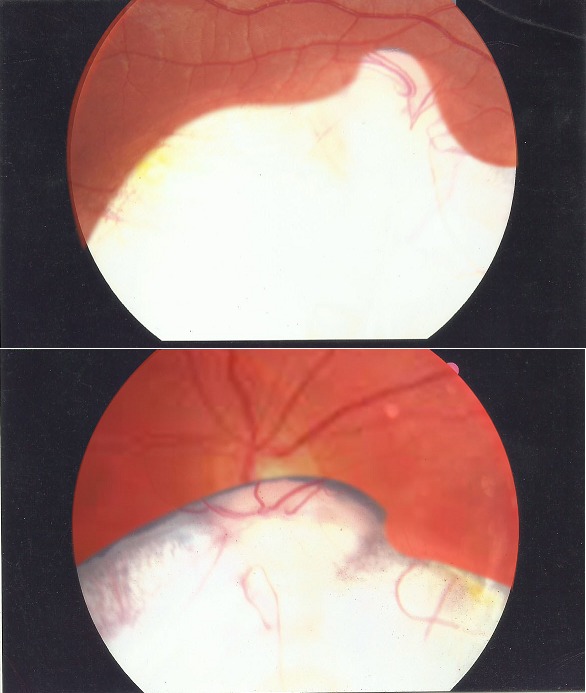
(A, B) colobome choriorétinien bilatéral, prenant toute la papille et plus de la moitié du pole postérieur du fond d’œil

Il s'agit d'un garçon de 6 ans amené par ses parents pour microphtalmie constatée depuis la naissance. L'examen ophtalmologique notait au niveau de l'œil droit une acuité visuelle à compte les doigts à 0,5 mètres avec une forte hypermétropie de +6 dioptrie. L'acuité visuelle n'étant pas améliorable par la correction optique du vice réfractif. Au niveau de l'œil gauche l'acuité visuelle est chiffrée à compte les doigts à 1 mètre avec une forte hypermétropie de +4 dioptrie non améliorable par la correction optique du vice réfractif. L'examen du segment antérieur note une microphtalmie au niveau des 2 yeux. L'examen du fond d'œil au niveau des 2 yeux notait un colobome choriorétinien très étendu allant de 3 heures à 10 heures englobant toute la papille Figure 1. Une échographie en mode B à été réalisée montrant la présence d'un nerf optique dans chacune des cavités orbitaires sans soulèvement rétinien associé. Cet examen à été complété par une tomodensitométrie orbito-cérébrale, un électrocardiogramme, une échographie cardiaque et une échographie rénale qui n'ont pas montré d'anomalies associées.

## Discussion

Les colobomes sont des malformations congénitales secondaires à un défaut de fermeture de la fente colobomique ou fœtale qui s'opère entre la 5^ème^ et la 7^ème^ semaine de la vie embryonnaire. Schématiquement on oppose les colobomes du segment antérieur à ceux du segment postérieur [4]. Dans notre étude le colobome choriorétinien était associé à une microphtalmie, cette association avait été évoquée par certains auteurs [5]. Si le colobome d'une part et la microphtalmie d'autre part sont souvent vus comme des entités pathologiques distinctes, certains auteurs ont émis l'hypothèse d'une origine génétique commune [6]. Dans notre cas il n'existait pas de décollement rétinien associé. Les colobomes choriorétiniens se caractérisent par leur extrême variabilité anatomo-clinique. En fin les colobomes peuvent être associés à plusieurs anomalies extra oculaires notamment d'ordre neurologique [7], imposant un bilan clinique et para clinique associé.

## Conclusion

Les colobomes papillaires peuvent revêtir plusieurs formes anatocliniques, cependant les formes associées à une microphtalmie sont rares. La conduite à tenir devant tout colobome reste la même, elle se base essentiellement sur une surveillance clinique et para clinique afin de dépister et de traiter précocement toute éventuelle complication.

## Conflits d’intérêts

Les auteurs ne déclarent aucun conflit d'intérêts.
